# An Optimized Strain-Compensated Arrhenius Constitutive Model of GH4169 Superalloy Based on Hot Compression

**DOI:** 10.3390/ma17143400

**Published:** 2024-07-09

**Authors:** Xiang Cheng, Ruomin Wang, Xiaolu Chen, Shasha Jin, Qinke Qian, He Wu

**Affiliations:** 1 Anhui Xinli Electric Technology Consulting Co., Ltd., Hefei 230601, China; chengxiang2083@163.com (X.C.); wangruoju@163.com (R.W.); akalune@outlook.com (X.C.); 18256977811@163.com (S.J.); 2 Key Laboratory for Light-Weight Materials, Nanjing Tech University, Nanjing 210009, China; 15851588327@163.com; 3 School of Electromechanical Engineering, Guangdong University of Technology, Guangzhou 510006, China

**Keywords:** hot deformation, flow behavior, constitutive modeling, GH4169 superalloy

## Abstract

A precise constitutive model is essential for capturing the deformation characteristics of the GH4169 superalloy in numerical simulations of thermal plastic forming processes. Hence, the aim of this study was to develop a precise modified constitutive model to describe the hot deformation behavior exhibited by the GH4169 superalloy. The isothermal cylindrical uniaxial compression tests of the GH4169 superalloy were carried out at temperatures of 950~1100 °C and strain rates of 0.01~10 s^−1^ using a Thermecmastor-200KN thermal–mechanical simulator. The original strain–stress curves were corrected by minimizing the effects of plastic heat and interfacial friction. Based on the true stress–strain curves, the original strain-compensated Arrhenius constitutive model was constructed using polynomial orders of 3, 5, and 10, respectively. The results showed that once the polynomial order exceeds the 5th, further increasing the order has little contribution to the accuracy of the model. To improve prediction ability, a higher precision Arrhenius constitutive model was established by extending a series of material parameters as functions that depend on temperature, strain, and strain rate, in which the error can be reduced from 4.767% to 0.901% compared with the classic strain-compensated Arrhenius constitutive model.

## 1. Introduction

GH4169 is a nickel-based superalloy widely utilized in the engines that work in the aerospace field and petroleum pipeline owing to its outstanding corrosion resistance, fatigue resistance, and high-temperature mechanical properties [1,2,3]. To enhance improved formability and service performance, the GH4169 alloy parts are primarily formed under thermal conditions [4,5], where the material flow behavior during thermoforming is very complex. Finite element simulation is widely recognized as an effective approach for process design and optimization. The accuracy of such simulations is contingent upon the constitutive model employed. Therefore, it is very necessary to develop an accurate constitutive model to predict the hot deformation behavior of the GH4169 superalloy.

At present, the constitutive model can be summarized into three categories: artificial neural network (ANN) models, physics-based models, and phenomenological models [6,7]. Based on the physics-based modeling approach, Tang et al. [8] established a group of constitutive equations based on internal state variables to investigate the evolution of microstructure and flow stress in Inconel 718, in which dislocations, grain size, and dynamic recrystallization (DRX) volume fraction were coupled into the model. This type of model has also been used to describe aluminum alloy [9,10,11], titanium alloy [12,13,14], and magnesium alloy [15,16,17,18]. However, this kind of model, consisting of a set of partial differential equations, has parameters that are difficult to directly determine and can only be obtained through inverse calibration methods [19]. The process of parameter solving is relatively complex. The ANN model, unlike mathematical models, does not rely on mathematical derivations. Instead, it focuses on training a model based on data without considering the underlying deformation mechanism [20,21]. However, the model is not propitious for further secondary development, making it impractical for applications in the forming process. Compared with both ANN models and physics-based models, phenomenological models are relatively simple. The typical ones include the Johnson–Cook (J-C) model [22] and the Arrhenius constitutive model [23]. The original J-C model posits that temperature softening, along with strain hardening and strain rate hardening, are distinct factors in the hot deformation process of materials. This simplifies the acquisition of model parameters but reduces predictive accuracy. Lin et al. [24] put forward an improved J-C model in which the interplays among deformation temperature, plastic strain rate, and plastic strain were considered. Sellars and McTegart [23] proposed an Arrhenius-type constitutive model that can express the influence of temperature and strain rate on deformation behavior through an exponential relationship with the Zener–Hollomon parameter. Yet, the original Arrhenius-type model neglects the impact of strain while solely focusing on the effects of strain rate and temperature, resulting in lower predictive accuracy [25]. Thus, a strain-compensated Arrhenius model was proposed to enhance the predictive accuracy of the standard Arrhenius model. In this model, several parameters are fitted to accurately describe their relationship with strain using a polynomial function [26,27]. Geng et al. [28] found that for linear friction welding on the GH4169, the predictive accuracy of the strain-compensated Arrhenius model is higher than that of the J-C model when comparing the experiments. In the strain-compensated Arrhenius model, the parameters are solely a function of strain and are independent of temperature and strain rate. However, numerous studies have indicated that deformation activation energy (*Q_act_*), which reflects the processability of the material and is closely linked to the thermodynamic mechanisms of dislocation motion, is influenced by both temperature and strain rate [29,30,31]. Thus, this means that expanding the *Q_act_* as a function dependent on strain rate, temperature, and strain within the strain compensation model can further enhance the accuracy and rationality of the model.

In this work, the thermal cylindrical uniaxial compression tests were taken to investigate the thermal flow and microstructure evolution of the GH4169 superalloy under temperatures of 950~1100 °C and strain rates of 0.01~10 s^−1^. Subsequently, the stress–strain data were corrected by excluding the effects of friction and heat generated from plastic deformation. The impact of polynomial fitting order on the strain-compensated Arrhenius constitutive model was investigated. Then, an optimized strain-compensated Arrhenius constitutive model was established by expanding the *Q_act_* as a function of temperature, strain rate, and strain. These results can offer valuable insights for describing and improving the thermal deformation process of the GH4169 superalloy.

## 2. Material and Experiment

**Figure 1:**
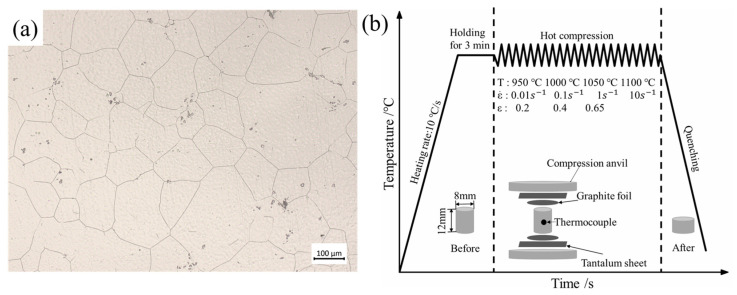
(**a**) Initial microstructure. (**b**) Experimental procedure diagram of thermal compression.

**Figure 2:**
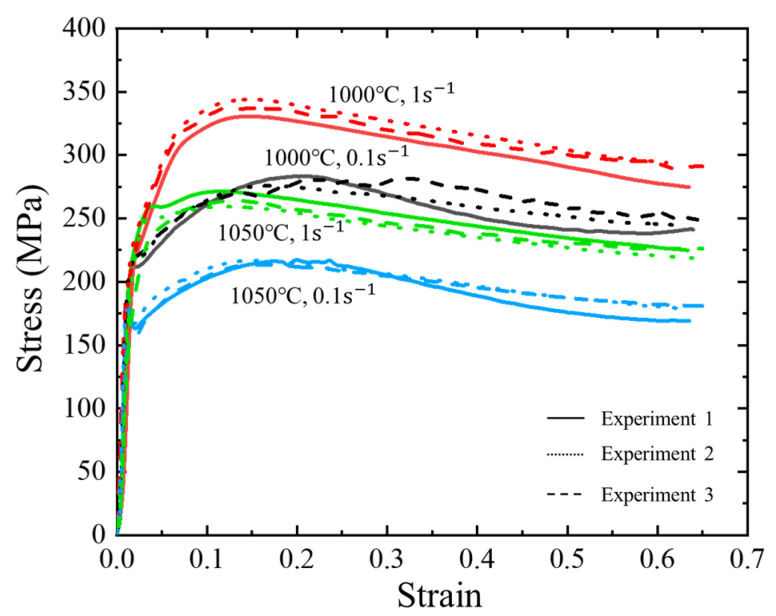
True stress–strain curves of GH4169 superalloy.

The composition (in weight percent) of the GH4169 alloy utilized in this investigation is outlined as follows: 50Ni, 17Cr, 4.75Nb, 3Mo, 0.6Ti, 0.5Al, 0.35Mn, 0.35Si, 0.2Co, 0.08C, 0.015P, 0.015S, with the remaining balance Fe. The initial microstructure is depicted in Figure 1a. The sample was subsequently manufactured into a cylindrical specimen measuring 12 mm in height and 8 mm in diameter. Thermal compression experiments were then performed using a Thermecmastor-200KN thermal–mechanical simulator (Manufactured by Fuji Denpa Koki Co., Ltd., Tokyo, Japan) at temperatures of 950~1100 °C and strain rates of 0.01~10 s^−1^. The equipment parameters/indicators are shown in Table 1. The lubricant and graphite foils were attached to minimize friction between the indenter and the sample. To enable temperature monitoring and feedback, a thermocouple was attached at the center of the sample. The sample was heated to the desired temperature of 10 °C/s and held at that temperature for 3 min to ensure uniformity. Subsequently, the samples were compressed to a true strain of 0.65 and immediately quenched to room temperature. The experimental procedure diagram is depicted in Figure 1b. In this study, the experiments were repeated three times, as shown in Figure 2. It can be seen that the experiment has good reproducibility. Therefore, in the subsequent analysis, we selected one set of data for analysis.

For microstructure analysis, all compressed samples were cut along the axial centerline using electrical discharge machining. Subsequently, mechanical polishing was performed, followed by etching in a solution of 5 g oxalic acid and 100 mL of water at 4 V for 10 s. The microstructure observation was carried out using an optical microscope(Olympus-BX53M). The recrystallization volume fraction was determined by performing statistical analysis using the Image-Pro Plus7.0 software.

**Figure 3:**
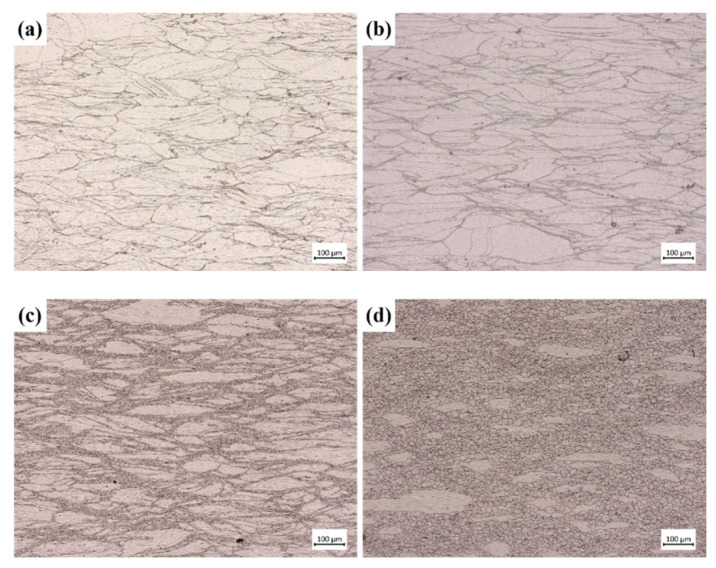
Microscopic structure of GH4169 at different temperatures of 0.1 s^−1^: (**a**) 950 °C; (**b**) 1000 °C; (**c**) 1050 °C; (**d**) 1100 °C.

Figure 3 shows the microstructure of the GH4169 superalloy at different hot compression parameters. It is evident that an increase in temperature has a simultaneous effect on the recrystallization grain size and recrystallization volume fraction. This is because high temperatures accelerate dislocation movement and facilitate dislocation climbing and annihilation, thereby promoting dynamic recrystallization.

## 3. Results and Discussions

### 3.1. Correction of Flow Stress

**Figure 4:**
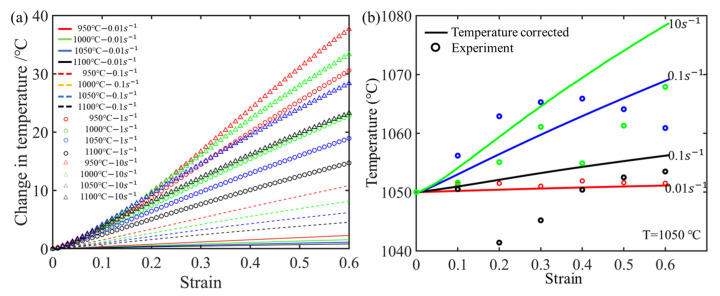
(**a**) Changes in temperature under different deformation conditions. (**b**) The comparison between the calculated temperature and the measured temperatures.

In hot compression experiments, deformation heating and friction have an impact on the strain–stress curve. These influences can result in errors in the experimental stress curve, thereby affecting the accuracy of the subsequently constructed model [21]. Therefore, the influence of temperature and friction on the stress–strain curve should be corrected. First, the corrections for temperature were applied to the experimental flow stresses. In the experimental process, the real temperature $T_{\mathrm{real}}$ measured using the thermocouple is typically composed of two components: the designated temperature for the experiment T and the additional temperature rise ΔT caused by deformation heating [32,33]

(1) $$T_{\mathrm{real}}=T+\Delta T=T+\frac{0.95\eta \int_{0}^{\varepsilon }\sigma d\varepsilon }{\rho C_{p}},$$

where *ρ* is the density; *C_p_* is the specific heat; $\int_{0}^{\varepsilon }\sigma d\varepsilon$ is the mechanical work (area under the uncorrected stress–strain curve); and *η* is the adiabatic correction factor related to strain rate. In this study, the values of *η* were set as 0.06 for a strain rate of 0.01, 0.2 for 0.1, 0.48 for 1.0, and 0.6 for 10.0 based on previous research reports [33,34,35] and our own experimental results. To mitigate the impact of Δ*T*, it is essential to first establish the relationship between temperature and stress. Figure 4a shows the changes in temperature under different deformation conditions based on the currently selected value of the adiabatic correction factor. It can be seen that the strain rate significantly affects the temperature increase of the sample. The higher the strain rate, the greater the increase in temperature. Moreover, the temperature rise decreases as the deformation temperature increases. To further validate the rationality of the selected value of the adiabatic correction factor, Figure 4b illustrates a comparison between experimental and calculated temperature increases at different strain rates under the condition of 1000 °C. The results indicate that the selected value for the adiabatic correction factor is reasonable.

To mitigate the impact of ΔT, it is essential to first establish the relationship between temperature and stress. The Arrhenius equation is widely employed to represent the correlation between temperature and flow stress, outlined as follows [23,36]:

(2) $$Z=\left\{\begin{array}{c} \begin{array}{c} A_{1}\sigma^{n_{1}}=\dot{\varepsilon }\exp(\frac{Q_{act}}{RT})\;(for\;lower\;stress\;level,\alpha \sigma <0.8)\\ A_{2}\exp(\beta \sigma )=\dot{\varepsilon }\exp(\frac{Q_{act}}{RT})\;(for\;higher\;stress\;level,\alpha \sigma >1.2) \end{array}\\ A[\sinh\left(\alpha \sigma \right){]}^{n}=\dot{\varepsilon }\exp(\frac{Q_{act}}{RT})\;(for\;all\;stress\;level) \end{array}\right.,$$

where *Z* is the Zener–Hollomon parameter; $Q_{act}$ is the deformation activation energy; R is the gas constant; T is the deformation temperature; and $A_{1}$, $A_{2}$, A, β, n, and $n_{1}$ are the material constants. By logarithmizing both sides of Equation (2), we obtained the following expressions:

(3) $$ln\dot{\varepsilon }=n_{1}ln\sigma +lnA_{1}-\frac{Q_{act}}{RT}$$

(4) $$ln\dot{\varepsilon }=\beta \sigma +lnA_{2}-\frac{Q_{act}}{RT}$$

(5) $$\ln[\sinh(\alpha \sigma )]=\frac{1}{n}\left(ln\dot{\varepsilon }-\ln A+\frac{Q_{act}}{RT}\right)$$

**Figure 5:**
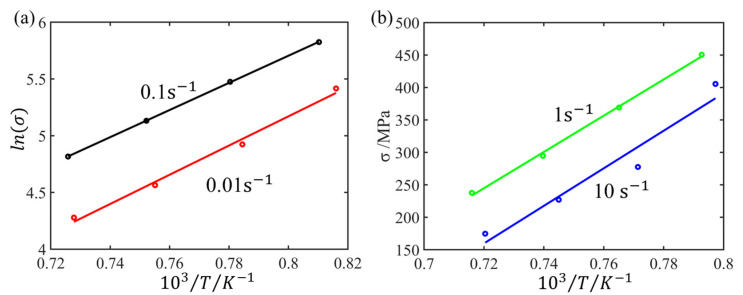
The linearly fitted relationship between (**a**) $ln\sigma -10^{3}/T$ and (**b**) $\sigma -10^{3}/T$ at strain 0.6.

**Figure 6:**
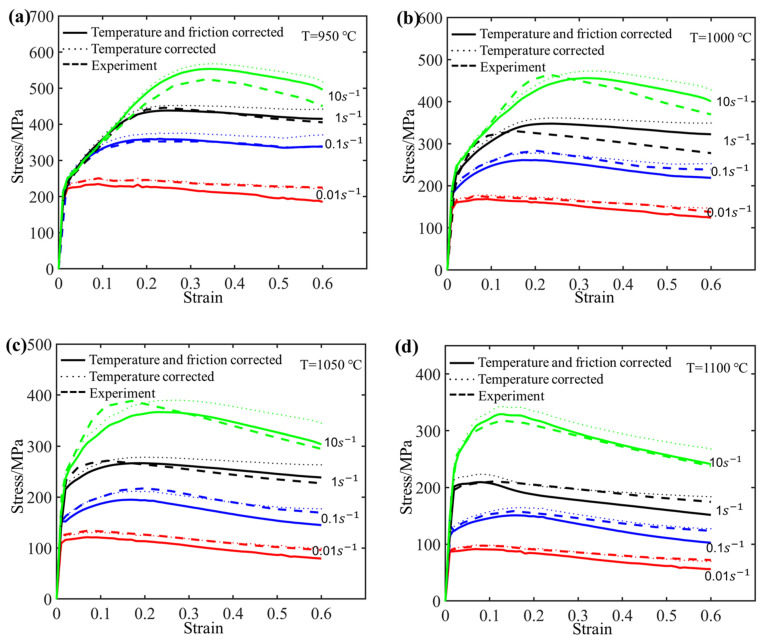
The comparison of stress–strain results between the temperature corrected and the double corrected and the uncorrected experimental flow stress.

At a specified strain value, linear regression analyses were performed for lnσ against 10^3^/T at low strain rates and σ against 10^3^/T at high strain rates, as illustrated in Figure 5. Utilizing the linear regression results for temperature and flow stress eliminates the effect of deformation heating on flow stress, resulting in the rectified flow stress at the predetermined experimental temperature, as illustrated in Figure 6. It is observed that the corrected temperature stress values are higher than the experimental stress values. This is consistent with the general expectation that higher temperature results in lower stress values.

**Figure 7:**
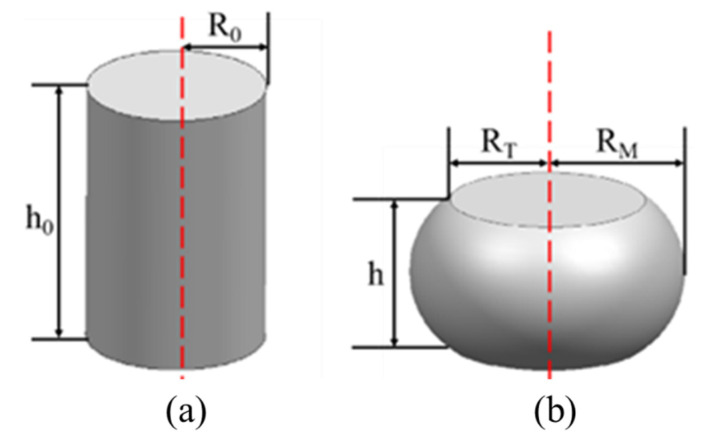
(**a**) The size of the sample before compression. (**b**) The size of the sample after compression.

In addition to the influence of deformation heating, the friction between the die and the workpiece can also lead to non-uniform deformation, thereby affecting flow stress. Despite attaching lubricant and graphite foils to minimize friction between the indenter and the sample, it is not possible to completely eliminate the influence of friction, as shown in Figure 7. It is observed that the specimen exhibits a bulging shape after hot compression due to the influence of friction. Therefore, the frictional correction of the shear stress is a necessary prerequisite for ensuring the precision of the constitutive model.

The friction correction Equation (6) was introduced to correct the impact of friction on experimental data [33]:

(6) $$\frac{\sigma_{0}}{\sigma }=\frac{8\mathrm{bR}}{H}\{[\frac{1}{12}+(\frac{H}{Rb}{)}^{2}{]}^{\frac{1}{2}}-(\frac{H}{Rb}{)}^{3}-\frac{me^{-b/2}}{24\sqrt{3}(e^{-b/2}-1)}\},$$

where *σ* is the corrected stress; *σ*_0_ is the uncorrected stress; *b* is the geometric shape parameters; *R* and *H* represent the instantaneous values of the radius and height, respectively; and m represents the level of friction. The m and b could be obtained as follows:

(7) $$\left\{\begin{array}{c} m=\frac{(R_{\mathrm{ave}}/h)b}{4/\sqrt{3}-(2b/3\sqrt{3})}\;\\ b=4\frac{R_{M}-R_{T}}{R_{ave}}\frac{h}{h_{0}-h}\; \end{array}\right.,\;\mathrm{with}\;\left\{\begin{array}{c} R_{ave}=R_{0}\sqrt{h_{0}/h}\;\\ R_{T}=\sqrt{3\frac{h_{0}}{h}R_{0}^{2}-2R_{M}^{2}}\; \end{array}\right.,$$

where $R_{ave}$, h, $R_{M}$, and $R_{T}$ are the average radius, height, maximum radius, and minimum radius of the deformed sample, respectively; $h_{0}$ and $R_{0}$ represent the height and radius of the initial sample, respectively. After the double correction of temperature and friction, the flow stress is shown as the solid line in Figure 6. According to the observation, the flow stress after friction correction generally exhibits a decreasing trend relative to the temperature correction. Additionally, as temperature declines and strain rate escalates, the differences between experiment and correction become more pronounced.

### 3.2. Original Strain-Compensation Arrhenius Constitutive Model

The Arrhenius constitutive model has been widely used due to its high prediction accuracy in high-temperature deformation [37,38,39]. In contrast to the original Arrhenius model, the strain-compensation Arrhenius model incorporates strain-dependent functions for its parameters α, $Q_{act}$, n, and ln⁡A [40]. Its fundamental form is shown in Equation (2). By using Equations (3) and (4), we can separately obtain the values of $n_{1}$ and β. Then, by utilizing $\alpha =\beta /n_{1}$, we can calculate the value of α in Equation (5). To determine the value of the activation energy of deformation, $Q_{act}$, the partial derivative of Equation (5) with respect to 1/T was taken. Then, $Q_{act}$ can be obtained as follows:

(8) $$Q_{act}=Rns=Rn{\left[\frac{\partial \mathrm{lnsinh}(\alpha \sigma )}{\partial (1/T)}\right]}_{\dot{\varepsilon }},$$

where n can be obtained by taking the partial derivative of Equation (5) with respect to $ln\dot{\varepsilon }$, i.e., $n={\left.\frac{\partial ln\dot{\varepsilon }}{\partial \mathrm{lnsinh}(\alpha \sigma )}\right|}_{T}$. As indicated by Equation (8), obtaining the values of n and s is a prerequisite for calculating $Q_{act}$.

**Figure 8:**
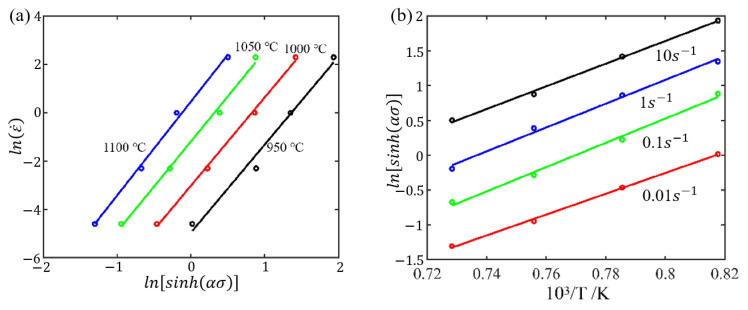
The linearly fitted relationship between (**a**) $ln\dot{\varepsilon }—\mathrm{lnsinh}(\alpha \sigma )$ and (**b**) lnsinh⁡(ασ)—1000/T at strain 0.6.

For instance, taking the linear fitting of $ln(\dot{\varepsilon })-ln[\sinh\left(\alpha \sigma \right)]$ at 0.6 strain, as shown in Figure 8a, the value of ${\left.n\right|}_{\varepsilon =0.6}$ can be obtained by calculating the average slope at different temperatures. Similarly, the value of ${\left.s\right|}_{\varepsilon =0.6}$ can be determined using the average slope of the linear regression of the $ln\left[\sinh\left(\alpha \sigma \right)\right]-1000/T$ at various strain rates, as shown in Figure 8b. Then, the ${\left.Q_{act}\right|}_{\varepsilon =0.6}$(=$R{\left.n\right|}_{\varepsilon =0.6}{\left.s\right|}_{\varepsilon =0.6}$) can be obtained.

Furthermore, logarithmizing both sides of the third equation in Equation (2) yields the following equation:

(9) $$\ln Z=n\ln[\sinh(\alpha \sigma )]+\ln A,\;\mathrm{with}\;Z=\dot{\varepsilon }\exp(\frac{Q_{act}}{RT})$$

**Figure 9:**
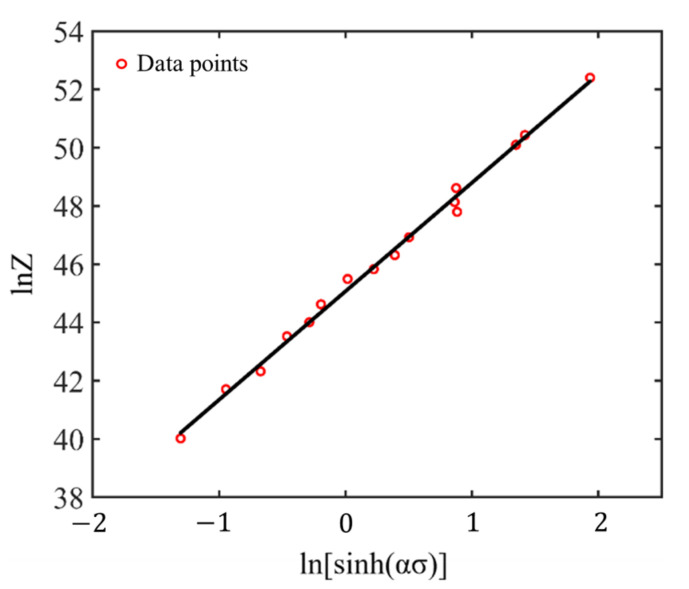
The linearly fitted correlation between lnZ−ln[sinh(ασ)] at strain 0.6.

Based on Equation (9), we can describe the linear fitting of ln⁡Z—ln[sinh⁡(ασ)]. Then, the slope ${\left.n\right|}_{\varepsilon =0.6}$ and intercept ${\left.\ln A\right|}_{\varepsilon =0.6}$ can be gained from fitting a straight line, as insulted in Figure 9.

**Figure 10:**
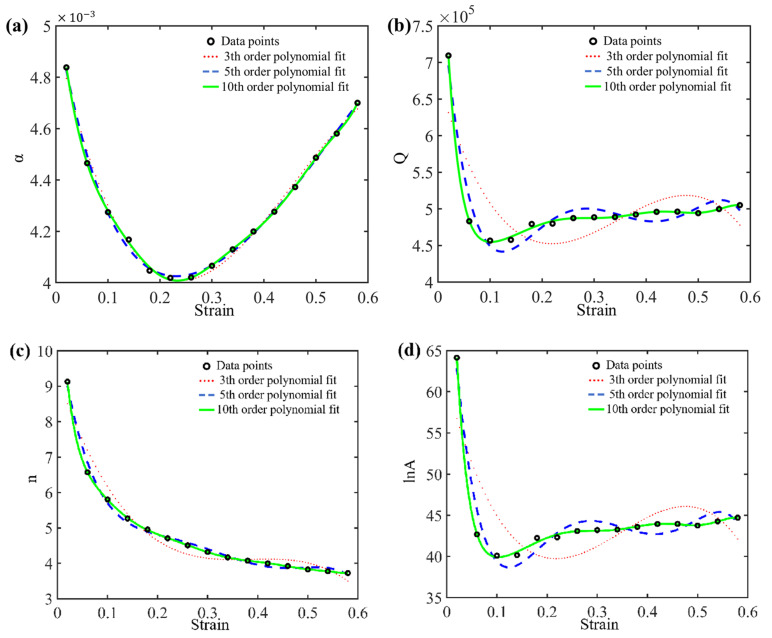
The n-order polynomial fitting curve of material parameters with strain: (**a**) *α*, (**b**) $Q_{act}$, (**c**) *n*, and (**d**) *lnA*.

In conclusion, by repeating the solving process with an interval of 0.04 within the strain range of 0.02–0.6, different values of the material parameters *n*, *Q_act_*, *A*, and *α* can be obtained for various strain levels. By using Equation (10), polynomial fitting can be performed to establish a function that describes the variation of material parameters with strain, as shown in Figure 10. Although there are some fluctuations during the fitting process, these are common fluctuations in the parameter polynomial fitting process. The fitting coefficients of polynomials of different orders are presented in Appendix A.

(10) $$\left\{\begin{array}{c} \alpha =\alpha_{0}+\alpha_{1}\varepsilon +\cdots +\alpha_{n}\varepsilon^{n}\\ Q_{act}=Q_{0}+Q_{1}\varepsilon +\cdots +Q_{n}\varepsilon^{n}\\ \begin{array}{c} n=n_{0}+n_{1}\varepsilon +\cdots +n_{n}\varepsilon^{n}\\ A=A_{0}+A_{1}\varepsilon +\cdots +A_{n}\varepsilon^{n} \end{array} \end{array}\right.$$

**Figure 11:**
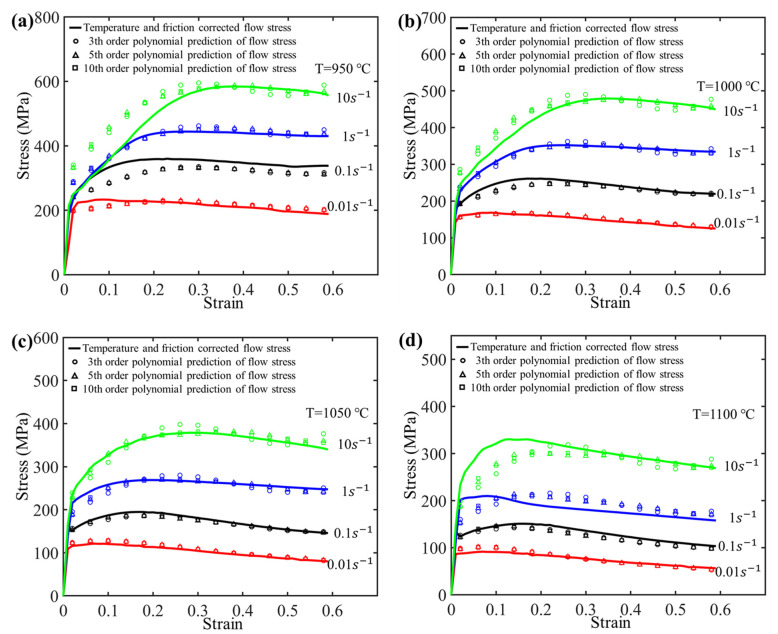
Comparing the experimental flow stress curves with the predicted ones that are calculated based on third-, fifth-, and 10th-order polynomials.

To investigate the impact of the fitting order on the model accuracy, parameter fitting using third-, fifth-, and 10th-order polynomials for the material parameters was conducted, respectively, as depicted in Figure 10. By substituting the calculated parameter values into the following equation, the predictive model for flow stress can be obtained, as shown in Figure 11.

(11) $$\sigma =\frac{1}{\alpha }arcsinh[exp(\frac{ln\dot{\varepsilon }-lnA+Q_{act}/RT}{n})]$$

As shown in Figure 10, the precision of the fits enhances with the increase in polynomial order. However, it can be observed from Figure 11 that the predictive accuracy of the flow stress improves only slightly when the polynomial fit of material parameters *n*, *Q_act_*, *A*, and *α* with respect to strain is increased from the third order to the fifth order. When the order of polynomial fit is further increased from the fifth order to the 10th order, the enhancement in model accuracy is considerably limited. To further enhance the predictive accuracy of the model, an optimized model is proposed in Section 3.3.

### 3.3. Optimized Strain-Compensated Arrhenius Constitutive Model

According to Section 3.2, it is evident that only increasing the polynomial fit order of the material parameters is not effective in improving the predictive accuracy. This is because the parameters in this model only take into account the influence of strain without considering the influences of strain rate and temperature during the deformation process. To enhance the predictive accuracy of the flow stress, this section introduces an optimized model that further incorporates the influence of temperature and strain rate into the model parameters. As illustrated by Figure 8a, the n values at different temperatures are distinct. Similarly, the s values at different strain rates also vary, as illustrated in Figure 8b. Therefore, n(ε) and s(ε) are extended to n(ε,T) and $s\left(\varepsilon ,\dot{\varepsilon }\right).$

**Figure 12:**
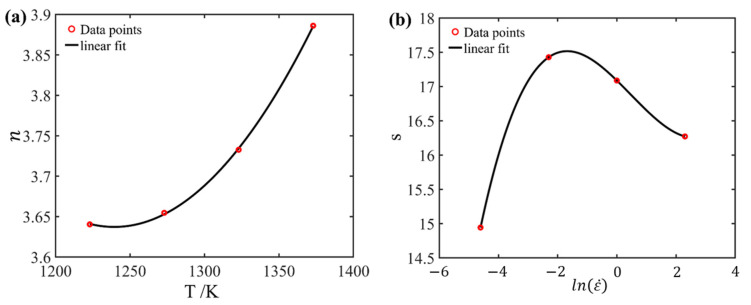
Fitting curve of n and s at true strain of 0.6: (**a**) n−T, (**b**) $s-ln(\dot{\varepsilon })$.

For the purpose of computing the values of n and s, an example at a true strain of 0.6 is illustrated here. Firstly, the n values at 1223 K, 1273 K, 1323 K, and 1373 K were computed as 3.640, 3.655, 3.733, and 3.886, respectively, based on Figure 8a, and the corresponding s values at 0.01 s^−1^, 0.1 s^−1^, 1.0 s^−1^, and 10 s^−1^ were calculated as 14.943, 17.428, 17.087, and 16.272, according to Figure 8b. Subsequently, polynomial fitting was performed for the *n* values at various temperatures and the *s* values at different strain rates. This method deviates from the traditional simple averaging approach, thereby ensuring higher accuracy for the values of *n* and *s*. Finally, the fitting results of n and s at a true strain of 0.6 are presented in Figure 12.

Additionally, $Q_{act}(\varepsilon )$ and lnA(ε) were expanded to $Q_{act}(\varepsilon ,\dot{\varepsilon },T)$ and $lnA(\varepsilon ,\dot{\varepsilon },T)$. As a result, the expression for the optimized model could be obtained:

(12) $$A(T,\dot{\varepsilon },\varepsilon )[\sinh(\alpha (\varepsilon )\sigma ){]}^{n(T,\varepsilon )}=\dot{\varepsilon }\exp(\frac{Q_{act}(T,\dot{\varepsilon },\varepsilon )}{RT})$$

The logarithm of both sides of Equation (12) was taken:

(13) $$\ln(\dot{\varepsilon })=n(T,\varepsilon )\ln[\sinh(\alpha (\varepsilon )\sigma )]+[\ln A(T,\dot{\varepsilon },\varepsilon )-\frac{Q_{act}(T,\dot{\varepsilon },\varepsilon )}{RT}]$$

Then, the $Q_{act}$ could be obtained by applying the partial derivative of strain rates to Equation (13) as follows:

(14) $$Q_{\mathrm{act}}(T,\dot{\varepsilon },\varepsilon )=Rn(T,\varepsilon )[\frac{\partial \ln[\sinh(\alpha (\varepsilon )\sigma )]}{\partial (\frac{1}{T})}{]}_{\dot{\varepsilon }}+\underset{P}{\underbrace{\left\{R\ln[\sinh(\alpha (\varepsilon )\sigma )][\frac{\partial n(T,\varepsilon )}{\partial (\frac{1}{T})}{]}_{\dot{\varepsilon }}+R[\frac{\partial \ln A(T,\dot{\varepsilon },\varepsilon )}{\partial (\frac{1}{T})}{]}_{\dot{\varepsilon }}-\frac{1}{T}[\frac{\partial Q(T,\dot{\varepsilon },\varepsilon )}{\partial (\frac{1}{T})}{]}_{\dot{\varepsilon }}\right\}}}$$

In general, the term of P was assumed to be zero [41,42]. Hence, $Q_{\mathrm{act}}$ can be simplified as follows:

(15) $$Q_{\mathrm{act}}(T,\dot{\varepsilon },\varepsilon )=Rn(T,\varepsilon )s(\varepsilon ,\dot{\varepsilon })$$

As discussed above, at each specific strain value, corresponding fitting curves of *n* with respect to *T* and *s* with respect to $\ln(\dot{\varepsilon })$ could be obtained. Based on this, the *n* and *s* curves at each strain value were extended in the strain dimension. For this purpose, *n* and *s* are described using the following polynomials:

(16) $$\left\{\begin{array}{c} \begin{array}{c} n\left(T,\varepsilon \right)=B_{1}\left(\varepsilon \right)T^{2}+B_{2}\left(\varepsilon \right)T+B_{3}\left(\varepsilon \right)\;\\ s(\dot{\varepsilon },\varepsilon )=C_{1}(\varepsilon )\ln(\dot{\varepsilon }{)}^{3}+C_{2}(\varepsilon )\ln(\dot{\varepsilon }{)}^{2}+C_{3}(\varepsilon )\ln(\dot{\varepsilon })+C_{4}(\varepsilon ) \end{array}\\ \begin{array}{c} B=a_{2-4}\varepsilon^{5}+b_{2-4}\varepsilon^{4}+\cdots +e_{2-4}\varepsilon +f_{2-4}\;\\ C=a_{5-8}\varepsilon^{5}+b_{5-8}\varepsilon^{4}+\cdots +e_{5-8}\varepsilon +f_{5-8}\; \end{array} \end{array}\right.$$

To establish a functional relationship between lnA and deformation conditions, the following steps were taken: firstly, $Q_{\mathrm{act}}$ was obtained using Equation (15); then, lnA was calculated using Equation (13); finally, a polynomial fitting was performed on lnA using ε, T, and $\ln(\dot{\varepsilon })$. The form of the polynomial is as follows:

(17) $$\left\{\begin{array}{c} \mathrm{lnA}(T,\dot{\varepsilon },\varepsilon )=D_{1}(T,\varepsilon )\ln(\dot{\varepsilon }{)}^{3}+D_{2}(T,\varepsilon )\ln(\dot{\varepsilon }{)}^{2}+D_{3}(T,\varepsilon )\ln(\dot{\varepsilon })+D_{4}(T,\varepsilon )\\ D\left(T,\varepsilon \right)=E_{1}\left(\varepsilon \right)T^{2}+E_{2}\left(\varepsilon \right)T+E_{3}\left(\varepsilon \right)\;\\ E=a_{9-24}\varepsilon^{5}+b_{9-24}\varepsilon^{4}+\cdots +e_{9-24}\varepsilon +f_{9-24}\; \end{array}\right.,$$

where $a_{i},\;b_{i},\;e_{i},$ and $f_{i}$ are the fitting coefficients, as shown in Appendix B. Since the parameter calibration process for the strain-compensated Arrhenius-type constitutive model involves a fitting process rather than solving equations for unknowns, the solution obtained is not unique. Some related studies have used the Genetic Algorithm (GA) to determine the model’s parameters. However, given that the predictive error of the parameters obtained using the current method was 0.901%, it is unnecessary to further optimize the parameters using the GA algorithm.

**Figure 13:**
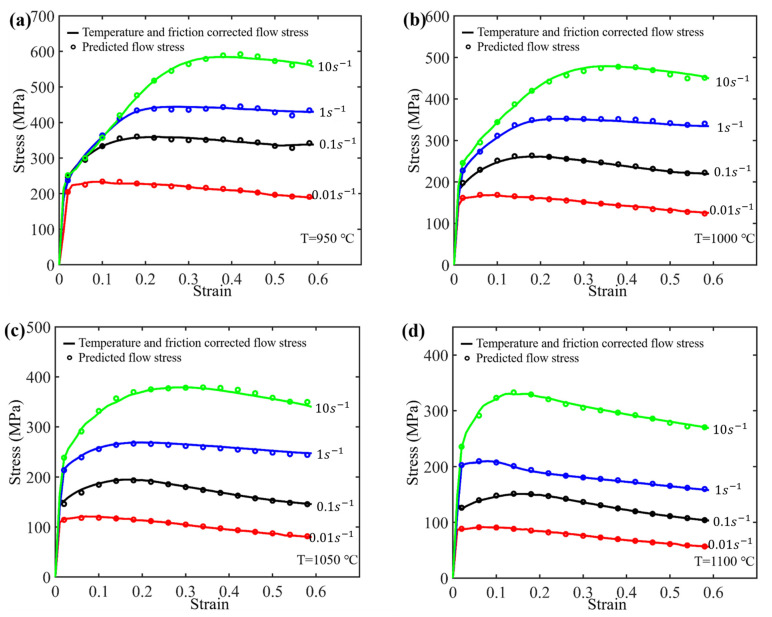
Comparing the experimental flow stress curves with the predicted ones that are calculated based on the optimized strain-compensated Arrhenius constitutive model (**a**) 950 °C, (**b**) 1000 °C, (**c**) 1050 °C and (**d**) 1100 °C.

By substituting the calculated parameter values into the above equations, the predictive model for flow stress can be obtained, as shown in Figure 13. It can be observed that, in comparison to the original model, the predicted flow stress of the optimized model is much closer to the experimental curve.

It is worth noting that the parameters in such a strain-compensated Arrhenius-type constitutive model are obtained through polynomial fitting, and all studies indicate that the coefficients used for this polynomial fitting do not have physical significance. The optimized model is an improvement on the strain-compensated Arrhenius constitutive model. Parameters with physical significance include $Q_{\mathrm{act}}$ and *ln*(*A*) in the Arrhenius equation, while the other parameters are fitting parameters for $Q_{\mathrm{act}}$ and *ln*(*A*). This is similar to the polynomial parameters used in all current research on the strain-compensated Arrhenius constitutive model and, therefore, similarly, does not have physical significance.

### 3.4. Correlation Analysis of Model Accuracy

In this section, a quantitative analysis is conducted to evaluate the accuracy of various models. As shown in Figure 11, for the original strain-compensation Arrhenius constitutive model, when the polynomial fit of the material parameters *n*, *Q_act_*, *A*, and *α* with respect to strain is increased from the third order to the fifth order, the predictive accuracy shows a slight improvement. However, as the order of polynomial fit increases further from the fifth order to the 10th order, the improvement in model accuracy is quite limited. Compared with the original strain-compensation Arrhenius constitutive model, the predicted results from the optimized strain-compensation Arrhenius constitutive model approach the target values, as shown in Figure 13. This suggests that the optimized model exhibits higher predictive accuracy.

For a more comprehensive evaluation of the precision exhibited by the diverse models, metrics like the correlation coefficient (*R*) and average absolute relative error (*AARE*) were introduced.

(18) $$R=\frac{\sum_{i=1}^{N}\left(E_{i}-\bar{E})(P_{i}-\bar{P}\right)}{\sqrt{\sum_{i=1}^{N}(E_{i}-\bar{E}{)}^{2}\sum_{i=1}^{N}(P_{i}-\bar{P}{)}^{2}}},$$

(19) $$AARE=\frac{1}{N}\sum_{i=1}^{N}\left|\frac{E_{i}-P_{i}}{E_{i}}\right|\times 100\%,$$

where $E_{i}$ and $\bar{E}$, respectively, represent the corrected flow stress and the average value of $E_{i}$; N represents the total number of selected data points used to assess the precision; and $P_{i}$ and $\bar{P}$ are the predicted flow stress and the average value of $P_{i}$, respectively.

**Figure 14:**
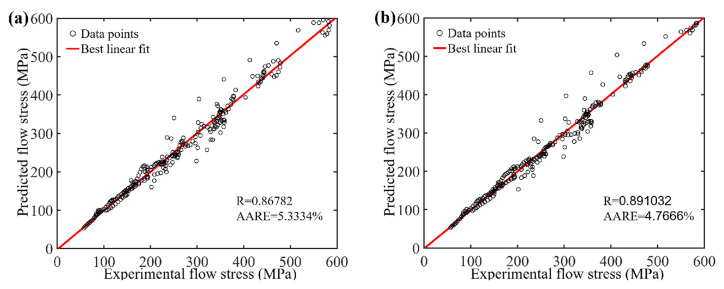

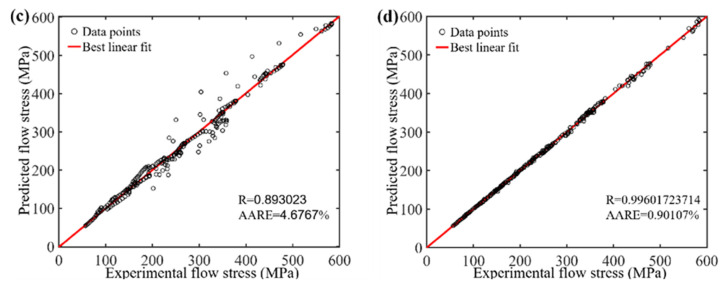
Correlations between experimental and predicted values calculated by original strain-compensation Arrhenius constitutive model with three different order polynomials and optimized model: (**a**) third-order polynomial, (**b**) fifth-order polynomial, (**c**) 10th-order polynomial, and (**d**) optimized strain-compensated Arrhenius constitutive model.

As shown in Figure 14, the slope of the red line represents the *R*-value, approaching one, indicating a strong linear correlation. Furthermore, an *AARE* value approaching zero implies a minimal discrepancy between the predictions and objects under comparison. It can be seen that the *AARE* value of the original model decreases as the polynomial fitting order increases, ultimately converging to a stable value of approximately 4.6%. Similarly, the *R*-value follows a similar pattern, stabilizing around 0.89. As for the optimized model, its *R*-value was 0.99602, with an error of 0.90107%, indicating a significant enhancement in precision compared with the original model. This means that expanding the *A* and *Q_act_* as a function of temperature, strain rate, and strain within the original strain compensation model can further enhance the accuracy and rationality of the model.

## 4. Conclusions

In this research, the thermal cylindrical uniaxial compression experiments of the GH4169 superalloy were taken at temperatures of 950~1100 °C and strain rates of 0.01~10 s^−1^. The flow characteristics of the GH4169 superalloy during thermal deformation were analyzed using the constitutive model. For the original strain-compensated Arrhenius constitutive model, once the polynomial order exceeds 5th, further increasing the order made a small contribution to the accuracy of the model. This is because the error in this condition mainly comes from the influence of temperature and strain rate on the model parameters under the same strain. For this reason, the optimized strain-compensated Arrhenius constitutive model is proposed by expanding the parameters (*α*, *n*, *Q*, *lnA*) as a function of temperature, strain rate, and strain. Compared with the original strain-compensation Arrhenius constitutive model, the optimized model shows an improved *R*-value, which increased from 0.89103125 to 0.9960172, and a reduced *AARE* value, which decreased from 4.767% to 0.90107%. Error analysis indicates that the optimized model exhibits better accuracy.

## Figures and Tables

**Table 1:** The equipment parameters/indicators.

Parameters	Value
Load Range/Accuracy	±200 kN, ±1%
Displacement Range/Accuracy	0~100 mm, ±1%

## Data Availability

The original contributions presented in the study are included in the article, further inquiries can be directed to the corresponding author.

## Appendix A. The Polynomial Fitting Coefficients of the Material Parameters for the Original Model

**Coefficients**	**α (0−n)**	**n (0−n)**	**$Q_{\mathrm{act}}\;(0-$n)**	**A (0−n)**
3th	0.004965196	9.280570422	676,621.0208	61.0612994
	−0.008999469	−40.86635503	−2,416,658.901	−230.8685229
	0.025313495	106.8353812	8,052,427.794	771.8335009
	−0.01837128	−92.3699141	−7,717,886.816	−741.5176644
5th	0.005043017	10.65750559	851,455.5084	77.65322329
	−0.011771629	−93.36139773	−9,118,905.766	−866.6237652
	0.049534267	606.0907809	72,199,014.41	6853.38013
	−0.100144531	−1958.54772	−249,146,829	−23,617.64701
	0.116324539	3001.975228	391,240,586.1	37,048.76691
	−0.05799663	−1741.610813	−228,771,494.6	−21,649.74571
10th	0.00513226	13.09915635	1,056,592.429	96.91444953
	−0.016181833	−288.2087321	−24,479,868.57	−2310.771568
	0.054076086	5629.3284	434,752,169.4	41,019.16905
	1.392636525	−66,157.84616	−4,487,663,282	−424,334.2081
	−21.94232655	483,956.3678	29,790,292,108	2,826,909.655
	146.935357	−2,277,882.923	−1.31145 $\times \;10^{11}$	−12,493,210.83
	−550.3668352	7,001,432.601	3.84125 $\times \;10^{11}$	36,729,726.51
	1229.917378	−13,953,848.96	−7.36399 $\times \;10^{11}$	−70,662,954.04
	−1628.38703	17,364,107.77	8.84414 $\times \;10^{11}$	85,154,905.52
	1178.395447	−12,253,784.79	−6.02175 $\times \;10^{11}$	−58,175,711.96
	−358.8138813	3,743,222.516	1.7704 $\times \;10^{11}$	17,162,940.37

## Appendix B. The Polynomial Fitting Coefficients of the Material Parameters for the Optimized Model

**Coefficients**		**a1−24**	**b1−24**	**c1−24**	**d1−24**	**e1−24**	**f1−24**
α		−0.313619694713256	0.543895041466033	−0.365608618478928	0.1244497630481	−0.02113597209330	0.005446613330949
n	B1	−0.126045546843889	0.234069957657822	−0.166675838101146	0.05645151186931	−0.00901272163852	0.000554240987898
	B2	345.630700332918	−640.985856269958	456.118762468758	−154.651085847321	24.8258673316506	−1.54646736038328
	B3	−237,324.596980361	439,540.520537349	−312,548.202652688	106,077.946074500	−17,119.5043604220	1084.20283268688
s	C1	43.1475033224648	−40.0123640650615	−4.26044031008300	13.6452551703253	−3.90601274917559	0.242723453398392
	C2	−22.3573005923190	198.105013817509	−265.935515353085	132.390269772340	−25.2298674879718	1.05008484641301
	C3	−1467.40331359746	2388.46163132963	−1387.84039308468	320.523456631582	−15.1669809803312	−2.60984588780090
	C4	−1104.82670101620	1315.29350890874	−237.622706864870	−236.643968782035	114.107490552172	0.149386135111619
D1	E11	0.00708824746160676	−0.01227964163259	0.007967811834393	−0.00238078908168	0.00031636052193	−1.82873445226331 × 10^−5^
	E21	−18.6422491869782	32.3810738259777	−21.0881975759859	6.33417033649789	−0.8478940186192	0.0489621270946009
	E31	12,237.1724058837	−21,310.793140206	13,927.6760969287	−4203.83601094965	566.486707459597	−32.6861294572920
D2	E12	0.0311730275899159	−0.053265420177463	0.0338179549946556	−0.009715027096117	0.00118707475111	−5.72799756454774 $\times \;10^{-5}$
	E22	−83.0178403898485	142.214199816963	−90.6129272024957	26.1602820906068	−3.21821326828688	0.153899437145203
	E32	55,186.4048879887	−94,771.1226350173	60,588.1297553708	−17,571.4685968231	2174.21976876730	−103.017266005603
D3	E13	−0.01808461137684	0.0363133985525667	−0.0283640156211	0.01084820348329	−0.0020392894982	0.0001815477277889
	E23	43.3108458068339	−88.8607082597213	70.9716779462624	−27.7793987816668	5.34156271632478	−0.482964627846992
	E33	−25,567.3413267429	53,840.2516391468	−44,115.9545491695	17,710.4701986799	−3487.55465679182	320.692350036855
D4	E14	−0.08087684880229	0.133937089478043	−0.07840675472315	0.01760586052878	−0.00034838051414	−0.000226671745111
	E24	212.00572653823	−352.06420682219	207.016839072178	−46.9114717964426	1.05093407600229	0.627590021585192
	E34	−138,735.817194794	231,056.612708095	−136,473.601976111	31,192.7365911438	−769.345619601457	−435.745738873583
